# Challenges and supports towards the integration of ePortfolios in education. Lessons to be learned from Ireland

**DOI:** 10.1016/j.heliyon.2018.e00899

**Published:** 2018-11-30

**Authors:** Paige Poole, Martin Brown, Gerry McNamara, Joe O'Hara, Shivaun O'Brien, Denise Burns

**Affiliations:** EQI, The Centre for Evaluation, Quality and Inspection, DCU Institute of Education, Ireland

**Keywords:** Education

## Abstract

This paper reports on research derived from a one-year study on the integration of ePortfolios in education using Ireland as a case example. Through a series of interviews with school principals, teachers and members of the support service of the Department of Education and Skills in Ireland, this research explores the opportunities and challenges relating to the use of ePortfolios in Irish post-primary education. Evidence suggests that, whilst supports for the integration of ePortfolios are beginning to emerge, there are many unresolved issues. These include equity of access to broadband coupled with the disconnect between ePortfolios and the curriculum that need to be addressed prior to ePortfolios becoming a common feature of the Irish educational landscape. Although the findings are particularly relevant to the Irish context, they also have wider implications for other jurisdictions that are in the process of introducing ePortfolios into their education systems.

## 1 Introduction

For two decades, it has been recognised that incorporating Information and Communications Technology (ICT) into Irish schools poses ‘a major national challenge that must be met’ (Department of Education and Skills, Ireland (DES), 1997, 2). The DES has sought to meet this ‘national challenge’ with the publication of various policy frameworks, such as *Schools IT 2000* (DES, 1997) and, more recently, the *Digital Strategy for Schools* (DES, 2015). The *Digital Strategy* presents the clear intention to support the introduction of ePortfolios in Irish compulsory-level education. Indeed, an objective of the first theme in the *Digital Strategy* is to ‘promote the use of digital portfolios (ePortfolios) for primary and post-primary students’ (DES, 2015, 27). This is no surprise given the alleged benefits of ePortfolios in an educational setting such as enhancing learning, assessment and professional development (see for example, Barrett, 2010, Batson, 2011, Klenowski, 2000, Stefani et al., 2007).

Butler et al. (2013, 2) noted that a ‘long-term vision for education’ is needed so that students can learn and receive all that is required to succeed in today's environment. On the other hand, paralleling Mulkeen's (2003) criticism that one of the main reasons for the unsatisfactory progress towards the integration of ICT within the Irish primary and post-primary sectors in the early 2000s was that policies offered no guidance for teachers on how to integrate ICT successfully, the *Digital Strategy for Schools* sets out how ePortfolios should be incorporated into the school curriculum. Based on the experience of the EUfolio project (DES, 2015) and through collaborative efforts between the DES, the Teaching Council of Ireland and other relevant bodies, the actions to be taken to implement ePortfolios in compulsory education focus on the rather esoteric promotion of ePortfolio pedagogy and purpose. As Marshal and MacNair (2005) observed, ‘the traditional debate on the use of portfolios has been on educational purpose (Meyer and Tusin, 1999), the embedding of reflection (Borko et al., 1997) and the demonstration of evidence (Klenowski, 2000), or any combination of these three interests’ (1). However, establishing a national, equitable ePortfolio system in compulsory-level education is more than a question of policy and promotion, not least because of the perceived financial burden associated with setting up and maintaining such a system. This is arguably because of what Buzzetto-More (2007), in reference to Tellefsen's (1995) constituent orientation analysis framework, calls the often fragmented ‘key constituencies’ associated with ePortfolio implementation (educators, learners, system designers, system managers). Indeed, as outlined by Pelgrum (2001), the embedding of any component part of ICT in education involves many factors and includes many variables that need to be considered. Furthermore, and adding complexity to the place of ePortfolios in education, there is also a wide range of interpretations around what comprises an ePortfolio, such as purpose and format, as well as around the tools that are used. Indeed, according to Hallam et al. (2008) there is no uniform definition of ePortfolio.

This paper provides an analysis of ‘key constituencies’ perspectives on the purpose of and subsequent barriers to using ePortfolios in post-primary education using Ireland as a case example. The first section of the paper analyses the literature on the functions of ePortfolios, the barriers to their implementation and the ePortfolio supports available to Irish Schools. The next section describes the methodology used in the present study. Then comes an analysis of the many voices and perspectives in this area, namely, a sample of those of school principals, teachers and members of the school support services at the Department of Education and Skills (DES). The last section presents the key findings, conclusions, and recommendations concerning the integration of ePortfolios in Irish post-primary education arising from the study.

### 1.1 Deconstructing ePortfolios in education

ePortfolio as a concept originates from its predecessor, the ‘traditional paper-based portfolio’ (Cyprus Pedagogical Institute, 2015, 10). Arguably both traditional portfolios and ePortfolios share common educational purposes such as storage, showcasing and assessment, while having differing characteristics (Cyprus Pedagogical Institute, 2015), with one of the most obvious difference being the digital benefit of ePortfolios when it comes to administering data (Fitch et al., 2008). Moreover, there are some portfolio definitions that are specific to their context (Gibson, 2006, 136) and with the rise of cloud-based storage have also developed over time (see, for example, Chang et al., 2014).

For example, Paulson et al. (1991) define portfolios as predominantly having a storage and showcase function: ‘A portfolio is a purposeful collection of student work that exhibits the student's efforts, progress, and achievements in one or more areas. The collection must include student participation in selecting contents, the criteria for selection, the criteria for judging merit, and evidence of student self-reflection’ (60). Wolf and Siu-Runyan (1996, 31) highlight that portfolios can also be used to showcase an ‘advancement of student learning’. Barrett (2010) describes the ePortfolio as ‘an electronic collection of evidence that shows your learning journey over time. ePortfolios can relate to specific academic fields or your lifelong learning. Evidence may include writing samples, photos, videos, research projects, observations by mentors and peers, and/or reflective thinking’ (6). It is possible to argue therefore, based on this emerging understanding of the potential offered by emerging technologies, that ePortfolios ‘go beyond the limits of paper-based portfolios’ (Theodosiadou and Konstantinidis, 2015, 18) and can be seen as reforming portfolios in order ‘to enrich students’ learning experience’ (DES, 2008, 2). Similarly, while also recognising the increasing use of cloud storage and dynamic workspaces that allow users to share and simultaneously collaborate on digital content, EUfolio (2015a) offers a contemporary definition of an ePortfolio as a tool that allows students to store, reflect upon and showcase their work.

ePortfolios are student-owned, dynamic digital workspaces wherein students can capture their learning and their ideas, access their collection of work, reflect on their learning, share it, set goals, seek feedback and showcase their learning and achievements. (8)

However, as ePortfolios serve different purposes they inevitably fall into different categories, such as ‘developmental, assessment and showcase portfolios’ (Regis University, 2003), depending on the way in which they can further a student's journey and on the curriculum specifications and assessment requirements of an education system. For Barrett (2010, 7), ePortfolios may be used for the purpose of ‘Showcase’ or ‘Accountability’ and ‘Learning or Reflection’. Thus, for a national ePortfolio system to be implemented, its purpose and how it links to curriculum specifications need to be explicitly stated. Barrett (2010, 8) highlights that ‘most ePortfolio systems tend to emphasize the showcase (portfolio as product) rather than the workspace (portfolio as process)’ function of ePortfolios. However, this represents an idealistic concept rather than the primary aim, which is to ultimately ‘shift from course-centered learning to student-centered learning’ (McCloud, 2004, 6). ePortfolios are not merely repositories for storing artefacts but can also serve to showcase work and to support assessment.

#### 1.1.1 ePortfolios as storage

Chang et al. (2014, 188) noted that ePortfolios can be accumulated and conserved through both ‘the Internet and cloud storage’, and Barrett (2010, 9) remarked that they can also be ‘stored on a server ... locally’. Thus, ePortfolios perform their function of storage in diverse ways, which naturally requires a robust ICT infrastructure. Ochola et al. (2015, 16) emphasised that this ‘invaluable infrastructure’ is a key criterion for the ‘successful implementation of an ePortfolio framework’ (Ochola et al., 2015). A robust system is obviously necessary for the storage of artefacts, but teachers and students will need to adapt to this new mode of evidence-based storage and consideration must also be given to their capacity to collect, store, upload and download artefacts (EUfolio, 2015a). Indeed, it is also evident that teachers will develop new roles (McGhee and Kozma, 2001) and will be required to pass on their knowledge to guide students ‘on the types of artefacts to save’ (Barrett, 2010, 9).

#### 1.1.2 Eportfolios as showcase

Stefani et al. (2007, 18) explained that ePortfolios display ‘a collection of the highest quality work that the student is capable of, and typically shows the range of work, perhaps with an idea of progression over time’. Students ‘showcase their reflections and achievements as well as contributions and feedback from peers and teachers’ (EUfolio, 2015a, 13). ePortfolios, therefore, have the potential to foster ‘reflective learning’ (Roberts et al., 2005, 7). However, as with any digital artefact, standards and the acceptable use of ePortfolios need to be made explicit in a school or organisation's acceptable usage policy (AUP). As with all forms of digital media, this, according to the Consortium for School Networking, will ‘protect students from harmful content on the internet’ and ‘provide students with good access to digital media to support engaged learning’ (2013, 2). Indeed, as with the storage function of ePortfolios, through exemplars of ePortfolio integration within existing ICT AUPs, it is important that teachers and students are educated on the legal requirements of and ramifications relating to the storage and showcase of digital content.

#### 1.1.3 Eportfolios to support assessment

Whereas summative assessment is often associated with end-of-topic testing ePortfolios for summative assessment link with their first purpose, which is the storage of student artefacts (EUfolio, 2015b). On the other hand, ePortfolios for formative assessment are centred on a ‘collaborative, continuous discourse between teacher and student’ (EUfolio, 2015b, 5) and as with all forms of formative assessment can be used to revisit the teaching and learning processes to accommodate student needs (Black and Wiliam, 2010). Indeed, Hattie and Timperley (2007) argue that ePortfolios are important drivers of continuous feedback, suggesting that ‘It is the feedback information and interpretations from assessments, not the numbers or grades, that matter’ (2007, 104). However, as with all forms of assessment for learning where an individual provides feedback, the primary participant responsible for the effective use of ePortfolios for formative assessment is the teacher with his or her ability to ‘promote learning’ through effective feedback (Rate, 2008, 22). This brings into question teachers' understanding and capacity to provide effective feedback for improved learning. In this context, the chief inspector for schools (Hislop, 2015, 14, in reference to Hattie, 2008) noted, ‘It is also self-evident that this sort of environment and teaching requires adequate teaching resources in each school, but also investment in the quality and ongoing professional development of teachers’. Thus ePortfolio deployment in education not only requires a robust ICT infrastructure and ePortfolio standards for showcasing students' work but also relates to ‘pedagogy and technology’ (EUfolio, 2015a, 18), which enable ePortfolios to be implemented effectively in the classroom and to be supported by the appropriate technology. To truly harness the benefits of ePortfolios in education, their deployment needs also to promote capacity building in the context of providing effective feedback for improved learning. As Cramer (1993, 72) observed, ‘the portfolio itself is not a type of assessment, but an assessment tool’.

### 1.2 Implementation of ePortfolios in education: challenges and opportunities

ePortfolio usage is inevitably difficult to implement over a short period, and challenges to ePortfolio implementation need to be addressed. Strudler and Wetzel (2005, 418) identified a number of these challenges highlighting issues ‘such as governance, leadership, and grants’. Lorenzo and Ittelson (2005) further specified the multiple issues that need to be explored before effective integration can take place, focusing on ‘hardware and software, support and scalability, security and privacy, ownership and intellectual property, assessment, adoption and long-term maintenance’ (8). Indeed, Young and Lipczynski (2007, 13) in reference to Love and Cooper (2004) identify common issues that need to be considered when designing ePortfolios for educational purposes.

1. The focus remains solely on the technical side rather than the administrative side;
2. ePortfolio is used as a content management system, rather than an interactive learning tool;
3. Stakeholders' views or needs are not included in the development of an ePortfolio;
4. ePortfolio is not fully integrated into the curriculum.

Although ePortfolios have been successfully implemented in component parts of some countries education systems such as England and Wales (see, for example, ElfEL, 2016), the literature on integration reveals significant challenges that need to be addressed. A study conducted in South Africa found that the most significant constraint on ePortfolio implementation was that, of the schools that participated in the study, only ‘10% indicated ready internet access’ (Kok and Blignaut, 2009, 5). Barriers to ePortfolio implementation also included not having a stable infrastructure, access, connectivity, socio-economic status and lack of integrated understanding of ICT, skills and confidence (Kok and Blignaut, 2009). In the absence of a stable ICT infrastructure it is no wonder, therefore, that implementing a system that is primarily technology-based is likely to be a problem for any education system that attempts to integrate ePortfolios into core teaching and learning practices. The EUfolio project also revealed barriers unrelated to the technological infrastructure of an ePortfolio ecosystem.

Citing the example of Cyprus, it reported on a situation where participants in an ePortfolio project voiced their concerns about the extra organisation required, the efficiency of their classes and the authenticity of student work (EUfolio, 2015b, 5). Slovenian and Lithuanian participants also expressed the worry that ePortfolios overemphasise the skills associated with their implementation, rather than the learning process itself (EUfolio, 2015b). Lithuanian and Cypriot teachers also stated that ‘there was a need to increase motivation for students’ work’ (EUfolio, 2015b, 10). This contrasts with Akçil and Arap's assertion that the use of ePortfolios by students will ‘motivate them to study’ (2009, cited in Yastibas and Cepik, 2015, 517). Thus, as an ePortfolio exists in an online virtual environment, it can be argued that its use will develop 21st-century skills. However, it is suggested that learning objectives and values be explored in detail with students before ePortfolios are implemented on a mass scale (Yastibas and Cepik, 2015, 515). In the Irish education context, barriers to the integration of ePortfolios mirrored those of their European counterparts. The EUfolio study found that Irish teachers were of the view that a significant amount of time was needed to assess ePortfolios effectively (EUfolio, 2015b), although one teacher stressed that, as with any new initiative, this became easier with practice (EUfolio, 2015b). Teacher Continuing Professional Development (CPD) is thus vital if ePortfolios are to be integrated into Irish education. Indeed, Baylor and Ritchie (2002, 4) similarly observed that any form of ICT integration requires training, as ‘technology will not be used unless faculty members have the skills, knowledge, and attitudes necessary to infuse it into the curriculum’. Spanish teachers also highlighted that there was a disconnect between ePortfolios and the curriculum, and the use of ePortfolios should be embedded in the curriculum in order to aid teachers with its establishment (EUfolio, 2015b, 16). This perspective also resonates with the disconnect between ICT and the Irish education system more generally. As stated by a member of the National Council for Curriculum and Assessment (NCCA) in Ireland.

The discussion around curriculum and assessment has tended to be exclusive of or has tended to exclude to some extent or not be connected with discussion around ICT – we are simply part of a trend in that regard…we see that trend shifting and changing (Johnston, 2014, 130)

There are, however, reasons to be optimistic. Despite the low priority accorded to investment in the knowledge economy during the period of sustained economic growth in the early 2000's (Austin and Hunter, 2013, 187) certain support networks and policy developments that will aid ePortfolio implementation are emerging in Ireland. For example, the *Digital Strategy for Schools* ‘sets out a clear vision for the role of ICT in teaching, learning and assessment for schools in Ireland’ (DES, 2015, 4) and acts as a blueprint to ‘promote the use of digital portfolios (ePortfolios) for primary and post-primary students’ (27). Moreover, as a result of Ireland's participation in the EUfolio pilot project, documents (such as EUfolio, 2013, 2015a, 2015d) have been produced to aid the successful incorporation of ePortfolios into post-primary schools. The *EUfolio Process Specification* document presents the work of the EUfolio team ‘for those who want to follow EUfolio's model’ (Department of Interactive Media and Educational Technologies, 2015, 3). The *EUfolio ePortfolio Implementation Guide for Policymakers and Practitioners* (EUfolio, 2015a, 5) also ‘illustrates the actions needed to introduce ePortfolios into schools’. Other ePortfolio assistance and specifications are also available to teachers in Ireland such as structured school support and a 5-hour online training session for teachers by the School support services of the DES – The Professional Development (PDST, 2015). In reality, however, although the EUfolio project was exploratory by design there is a noticeable absence of the voices of those key constituencies who are tasked with the implementation of ePortfolios, namely principals, teachers and members of the support service of the DES. This is significant as, to cite Coe (2009, 368), ‘if we hear only the success stories then pretty much anything may seem like an effective strategy for school improvement’. In the latter part of this paper we report on the insights and opinions of these key stakeholding groups with a view to integrating their experience and expertise into the analysis of the value and challenges of ePortfolio usage.

## 2 Methodology

The purpose of this study was to explore the challenges and opportunities towards the integration of ePortfolios in post-primary education using Ireland as a case example. In order to gather data a stratified purposeful sampling strategy was adopted (Patton, 2014). This saw researchers conduct interviews with key constituencies - educators, system designers and system managers (Buzzetto-More, 2007) - who are responsible for the deployment of ePortfolios in Irish education. The selection of participants was initially based on an equal distribution of educators (principals and teachers) who have responsibility for the integration of ePortfolios in their respective schools. However, the researchers also wanted to interview key stakeholders who could provide perspectives on the place of and barriers towards the integration of ePortfolios in Irish post-primary schools. For this reason, the previously mentioned stratified purposeful sampling technique was chosen in which members of the support service of the DES were also selected for interview. Indeed, as was the case in this study, Patton states, ‘the purpose of a stratified purposeful sample is to capture major variations rather than to identify a common core, although the latter may also emerge in the analysis’ (2002, 240). In this regard a series of semi-structured interviews were conducted with three distinct groupings.

**Group 1:** This group consisted of four members of the support services of the Department of Education in Ireland who are responsible for ePortfolio infrastructural and pedagogical school and system level supports in Ireland. Each group member is codified as DE1, DE2, DE2 and DE4.

**Group 2:** This group consisted of four Principals and Deputy Principals who are responsible for the deployment of ePortfolios in their respective schools. Each group member is codified as P1, P2, P3 and P4.

**Group 3:** This group consisted of four school teachers who are responsible for the integration of ePortfolios in their classroom practice. Each group member is codified as T1, T2, T3 and T4.

Each of these groups took part in a semi-structured interview process that lasted for between one and two hours. Participants were asked a series of questions relating to (1) the place of ePortfolios in Irish post-primary education and (2) the challenges and opportunities towards ePortfolio integration in Irish education.

Following on from this, using Creswell's (2008) data analysis process andMiles and Huberman's (1994) ‘Components of Data Analysis: Interactive Model’ emerging themes relating to the integration of ePortfolios in Irish education emerged. These themes are presented in the analysis section of the paper.

Ethical approval for this research was provided by the School of Education Studies, Dublin City University. All interviews were conducted according to established ethical guidelines, and informed consent was obtained from the participants.

## 3 Analysis

Consistent with previous research by Barrett (2010) and EUfolio (2013), key constituencies who were interviewed had similar views as to the structural requirements of an ePortfolio system that allows students to store and share work and also to transfer digital content from ePortfolios as they progress throughout their education and career paths. Participants were also positively disposed towards ePortfolios that could be used as a tool to significantly enrich teaching and learning. In light of significant changes to the curriculum requirements of the first phase of Post Primary education in Ireland (the Junior Certificate) that places a significant emphasis on key skills for the world of work such as communicating, being creative and working with others (National Council for Curriculum and Assessment, 2012); participants also believed that ePortfolios would allow for these key skills to become more achievable and manageable. The following part of this study is divided into two sections, covering the place of and structural requirements for ePortfolios in Irish education.

### 3.1 The place of ePortfolios in Irish post-primary education

Although participants had varying roles all agreed on the need to deploy ePortfolios in education. According to P1, ‘anything that enhances the learning experience would be of great benefit’. In line with the drive towards increased personalised learning (see, for example, McLoughlin and Lee, 2010) all participants thought that an ePortfolio system allows teachers to readily provide assessment feedback and consequently allows students to be able to record and reflect on their work. As T1 puts it, ‘ePortfolios have the ability to transform how I teach’. Consistent with Mann et al. (2009), who stress the importance of the education system to develop ‘reflective ability in their learners’ (596), T3 states:

The possibilities for ePortfolios are endless. I mean, in one location I will be able to provide assessment feedback to the student and challenge the student to reflect on what feedback has been received and then see if the feedback has been of any use. Did the student really benefit from what I was saying? Did their work actually improve?

Participants agreed on the benefits of ePortfolios as a tool to enrich assessment for learning practices. For example, DE3 stated that ePortfolios can be used for ‘all types of assessment, summative or formative’, and P1 observed that ePortfolios can be used for ‘projects, assignments and even things like basic homework’. P2 also noted their use as ‘a scaffolding tool for formative and summative assessment, and it [ePortfolios] can capture the quality of work they complete, and can be used to assess the learning taking place’.

Another participant, P4, also referred to ePortfolios as a core tool to develop Junior Cycle Key Skills (NCCA, 2012). ‘If you look at the Key Skills, Managing Myself, Managing Information and Thinking, Being Creative, etc., how this will play out in the classroom can be unnerving for teachers, but the various features of an ePortfolio can help to put a structure to all of this.’ P3 echoed this, stating: ‘A lot of the time I'm asked by staff how we're going to manage all of this, which is a fair question, but I think that ePortfolios can be used to make our lives a lot easier and using ePortfolios will make teaching easier and will also give students more responsibility for their learning, which is what I hear on a regular basis from parents and staff. I wish they'd take more responsibility…!!’ All respondents agreed that the most significant aspect of an ePortfolio system is its capacity ‘to enrich a student's learning experience’ (DES, 2008, 2). DE3 explained that ‘the process and ease of teaching and learning derived from an ePortfolio system should be the key message for schools and not the digital media or the product itself’. DE4 underlined this idea, saying: ‘A lot of the conversations I have heard [are about] using technology, which is the wrong way of looking at it for me.’ Indeed, DE3 condemned the input of ‘multi-nationals and software companies’, arguing that ‘they are pushing something to promote the product’. This reinforces Barrett's (2010) caution about ePortfolios being wrongly promoted as a product, as opposed to a functional tool for learning.

### 3.2 The structural requirements of an ePortfolio system in Irish post-primary education

Participants were also in agreement as to the core specifications of an ePortfolio system in education. T3 stated that ePortfolios should be transferable so that the ePortfolio ‘should travel with me throughout my education and into college or work’. P2 further stated that an ePortfolio should be ‘platform agnostic, in that it should be accessible on multiple devices’. DE3 said that the ePortfolio should be ‘accessible for all, so it does not have to be platform specific’. In DE2's view, an ePortfolio should be a ‘hybrid tool, evolving in the cloud that encompasses a Swiss army knife of tools’. T1 observed that an ePortfolio system should be ‘composed of various types of electronic and non-electronic media’. Furthermore, the storage, showcase and reflective features of ePortfolios were described as being essential requirements of student ePortfolios. DE1 explained that the storage capacity of an ePortfolio ‘can help manage digital files’. DE2 added that it helps ‘students to organise their learning into a space’. P2 also put forward the idea of ePortfolios being ‘cloud-based’, thus supporting the perspective of Chang et al*.* (2014) on the ever-changing nature of ePortfolios. Similarly, EUfolio (2015c, 6) states that ePortfolios should be available ‘any place, anytime and for any purpose’. Finally, the showcase function of an ePortfolio was also described by DE1 as having the ability to be ‘public in nature’, implying that students ‘share parts of their ePortfolio along their learning journey’. DE4 reinforced this point, stating that, upon showcasing their work and accepting feedback; students would be able to ‘make changes and adapt for reflection on feedback’.

### 3.3 Barriers to ePortfolio integration in Ireland

In the light of previous research on proposed specifications and the benefits of ePortfolios in Irish education, there appeared to be agreement among stakeholders on the barriers to the common use of ePortfolios in Irish education and specifically relate to: (1) the disconnect between the curriculum and ePortfolio functions; (2) teacher and student capacity; (3) internet connectivity; (4) clarity of ePortfolio ownership and (5) the device.

#### 3.3.1 The disconnect between the Irish curriculum and ePortfolio functions

While identified barriers were a significant finding in this study, certain factors were highlighted as being able to aid the development of an ePortfolio system. These included the defragmentation of government policies and possible entry points for an ePortfolio system. As stated by P1, ‘you cannot resist technology; you have to embrace it’. The integration of an ePortfolio system seemed to outweigh the barriers. All participants agreed that ideally ePortfolios should be implemented at all levels of compulsory-level education. DE1 commented that ‘by the time students reach post-primary level, their learning should be more profound’. In this way, by introducing ePortfolios at primary level, T3 is also of the view that ‘ePortfolios would only be seen as a tool for learning and nothing more’. If ePortfolios were to be used at primary level, then their use would foster the third aim of primary education, ‘to prepare the child for further education and lifelong learning’ (National Council for Curriculum and Assessment (NCCA), 2004, 1). Accordingly, DE3 expressed the view that ePortfolios ‘should be used from ABC to PhD.’

On the other hand, teaching and learning at post-primary level are circumscribed by the requirements of externally devised curriculum specifications and recommendations for effective assessment practices. P2 pointed to a continuing weakness in the Irish teaching model at senior cycle post-primary level, in that ‘it is content driven and factual, rather than explanatory and developmental’. P2 stated that ‘our [traditional] teaching model does not lend itself well to the ePortfolio model.' This suggests that for ePortfolios to be embedded into the Irish education system, there should be a realignment between the purpose of the ePortfolio and curriculum practice which is also in line with McCloud's (2004) advocacy of student centered learning and with the drive towards increased use of effective feedback within assessment for learning practices. Participants further raised concerns about the prescriptions of the curriculum and how they align with the various ePortfolio functions. DE2 provided a succinct summary of the doubts about the current education system adopting ePortfolios.

‘The curriculum has flexed up, but assessment hasn't’. (DE2)

T2 was also of the view that ‘ePortfolios [are] a nice thing to do’. However, the efficacy placed on ePortfolios is not particularly strong in the Irish education system

ePortfolios were always there but were not embedded because there was no need to, and I think it is time to move on from the idea of ePortfolios being something nice to have, but they are not that important. If we keep going this way, we can wipe out a good few years. ePortfolios have to become part of the requirements for assessment if you want them to be used.

#### 3.3.2 Teacher and student capacity

All participants outlined their concerns about teachers and students in relation to the integration of ePortfolios. Teacher confidence and the requirement for increased CPD were seen as a significant issue. In agreement with Baylor and Ritchie (2002), who thought that teacher training is required to integrate new technologies into classroom practice, teachers will, according to DE4, need technological upskilling, as ‘their expertise is not in this area’. Indeed, in T2's view,

all these things, live or die, you need to convince staff that ePortfolios will improve their own and their students' practice, and it will reduce not increase their workload. The one and only way that we can do this is in a very safe and scaffolded way. How does it benefit us as teachers? How does it benefit the student?

Similarly, DE4 observed that ‘if there is no buy-in from the teacher, then the whole thing falls.’ Indeed, according to T2, ‘all teachers young and old need a lot of CPD in this area. Everything from how ePortfolios benefit classroom practice, how to store files, how students’ work can be displayed, all the way through to how we can provide feedback to students.’

Although it is evident that CPD is required for all teachers on the use of ePortfolios, a significant finding in this study also related to student capacity. DE4, T1, and T3 agreed that there is a common misrepresentation of the ICT capacity of students.

There is a slight misconception that the digital native is very confident in using digital technology. While they might have the language, their proficiency levels are not where they need to be, to be proficient users of an ePortfolio. (DE4)

There is a lack of ICT/technology skills among the younger kids. I know they spend their time on iPads, but they do not have the skills, I think. (T1)

Yes, students know how to use social media very well. A lot even know how to use a word processor to type a CV. A few even know how to use Apps to create, but the majority of students in Ireland do not know how to store and organise digital files, showcase their work or even reflect on their work in a digital environment for that matter. (T3)

This suggests that students also need support, primarily from their schools. Miller and O'Neill (2014) supporting this argument asserted that teachers will be expected to aid students in the development and personalisation of their ePortfolios. In the same vein, Tosh et al. (2005) stated that ‘students have to know what an ePortfolio is, how to use one and, most importantly, how it may benefit them.’ Indeed, the significance of ePortfolios' being student-led is that they allow students to be ‘able to speak up about their education’ (Thomson, 2011). On the other hand, given the constraints of curriculum and assessment practices and how they relate to standardised test results; according to T2, ‘we all want the best for students but the demands on staff are high as they are already on CPD overload and some just don't see the point when students are still going to be judged on what they get in their Leaving Certificate [Senior Cycle] examination’.

DE1, emphasising the non-alignment between the Senior Cycle curriculum and ePortfolio functions, stated that, ‘although Junior Cycle reform is going through a contentious phase, reform of the Leaving Certificate and how students move to third level needs to be examined’. In P2's view teachers need to be encouraged, through the use of ePortfolios, to become ‘facilitators of knowledge’ as opposed to facilitating the pursuit of maximum outputs from preparing for high stakes examinations, which requires very little use of ePortfolios.

Another issue is related not so much to the procedural capacity of ePortfolios but rather to the requirements for upskilling on the use of ePortfolios for assessment for learning. According to T3, ‘The problem isn't only about upskilling teachers on how to store a file, showcase an electronic file. That's the easy part’. All participants agreed that teachers should be provided with training on effective assessment for learning and concurrently on how ePortfolios can be used as a tool for assessment.

#### 3.3.3 Internet connectivity

All participants agreed that a major obstacle to the introduction of ePortfolios in Ireland related specifically to external factors beyond their storage, showcase and process functions. As T3 noted, ‘The big problem isn't about providing CPD, the big issue is broadband and Wi-Fi access.’ P1 observed that ‘each school would have to have 100 Mbs broadband’, which concurs with figures from the Department of Communications, Energy and Natural Resources (2015) that ‘All 780+ post-primary schools in Ireland now have access to 100Mbps high-speed broadband’. By contrast, DE3 stated that ‘there is no equity to broadband access, particularly in rural areas.’ Indeed, according to DE1 ‘a lot of ePortfolio work will not take place during school hours, it will take place outside of these hours.’ Therefore, a strong ICT infrastructure must be available in schools and at home, reflecting the views of Lorenzo and Ittelson (2005) on the barriers associated with ICT integration in education. Indeed, Amarach Research confirmed that ‘One in four broadband users in rural Ireland or 450,000 people say their speed is too slow (rising to a third of all users in the countryside and 44% of those living in detached houses in the countryside)’ (2016, 13). Consistent with previous studies on barriers associated with ePortfolio implementation (see, for example, Kok and Blignaut, 2009), these stark figures on the need for equitable access to effective broadband speeds highlight the practical limitations of ePortfolios in Irish post-primary education, as well as the issue of equity. As T3 stated:

Our school is very limited in what we can do with ePortfolios. We have broadband, and we have Wi-Fi, which is fairly good. Most of the staff, their ICT skills are good and are very positive towards the use of ePortfolios, but it [ePortfolios] is limited to school hours, so there's not a lot that can be done with it apart from large-scale tasks. Asking students to continue any form of learning, doing homework, whatever it is, using ePortfolios brings up all sorts of problems and at present it is just not worth it.

#### 3.3.4 Clarity of ePortfolio ownership

It was generally agreed from DES support services that ePortfolios should be student-owned. DE1 outlined the benefits, stating that ‘the key stakeholders, students, are sometimes the voice that's missing’. ePortfolio ownership by students would also allow them to ‘take responsibility for their own learning’, as envisaged by the NCCA (2010, 10). DE2 confirmed that the creation of an ePortfolio should be primarily ‘student owned and selected’, allowing students to ‘choose what works for them’. Personally owned ePortfolios would allow for the decentralisation of ePortfolio maintenance away from the school and the DES and would put the responsibility for maintenance on the student instead. ‘If ownership is at a personal level, the administrative burden must be at a personal level too’ (DE1). DE2 was also of the opinion that, ‘since there are multiple entities out there… there is no maintenance on them [ePortfolios]’.

Interestingly contrary to the views of the support service personnel on ePortfolio maintenance, teachers proposed the opposite. P2 highlighted that ‘for economy and scale, it [maintenance] would have to be done at a national level’. T3 also expressed concerns relating to ‘the resources required to maintain the system locally’, reinforcing the view of P2 that ‘it would be hard to have someone in school doing this’. It is evident that Strudler and Wetzel's (2005) concerns about funding, ownership and creation are paramount among school personnel with regard to ePortfolio ownership. However, P1 declared that if an agency-maintained ePortfolio system existed, ‘the technological team in the school would have to participate in the creation of such a system’. T1 added that ‘teachers would need to be involved and also educational policy makers’. Similarly, T2 stated that ‘Teachers, technical companies, the State Examination Commission (SEC), the NCCA, PDST, JCT (Junior Cycle Support Service) and other support services should all be involved in forming a consensus on these issues.’

#### 3.3.5 The device

The device itself was also deemed to be an issue that needed further exploration among key constituencies. P2 expressed the need for ‘a reasonable suite of ICT devices’ in schools. DE2 also highlighted the need for students to bring their own device, the benefits being that ‘students should use their own device, and they can engage using their personal device that reflects their learning style.’ This would involve schools accommodating the ever-increasing growth of the *Bring Your Own Device* (BYOD) initiative (Downes, 2012). DE2 also acknowledged that ‘it is a challenge if all students use different tools’. T3 cautioned that ‘if BYOD was to become the norm’, it would be essential for the DES or its support service to ‘provide minimum technical specifications for BYOD that can facilitate the learning outcomes of the curriculum’. Indeed, according to DE4, ‘determining use is a key conversation that has to be had’. There was a consensus that policy-level recommendations, minimum specifications and standards should be agreed by the various key constituencies, such as the DES and those who are responsible for curriculum and assessment, namely the National Curriculum Council for Assessment and the State Examinations Commission.

## 4 Discussion and conclusion

This study explored the concept of an ePortfolio system from its origins to its place in education using Ireland as a case example. Semi-structured interviews with a range of stakeholders who have responsibility for the deployment of ePortfolios at a policy and school level yielded findings that resonate with the literature. On the one hand participants agreed on the structural requirements of an ePortfolio in Irish education and were unequivocally favourably disposed towards ePortfolios as a tool that can considerably enhance teaching and learning practices. On the other hand, participants were also of the view that challenges exist such as equity of access to broadband and the performance demands of the Senior Cycle curriculum would significantly prevent the true benefits of ePortfolios as a tool for learning from being exploited. Furthermore, they suggested that a personal ePortfolio system seemed to be preferred for a variety of reasons, such as the costs of maintaining the system, coupled with the view that ePortfolios should be transferable and dynamic workspaces.

Fullan observed (2007) that for any new initiative, ‘The total timeframe for initiation to institutionalization is lengthy; even moderately complex changes take from 2 to 4 years, while larger-scale efforts can take 5–10 years, with sustaining improvements still problematic’ (67). Taking this into account and excluding issues relating to broadband access, the following recommendations are made in relation to using ePortfolios as a tool to enhance teaching and learning across the continuum of post-primary education.

### 4.1 A common understanding of an ePortfolio

Before advances can be made in integrating ePortfolios into education, a common understanding of the purpose and function of ePortfolios as a tool for realising learning outcomes needs to be more explicitly stated. National agencies must collaborate to provide unified standards and specifications for the use of ePortfolios in conformity with the curriculum. This would have the benefit of allowing each school to adopt an ePortfolio system specific for its own context while at the same time providing a blueprint on how ePortfolios can be used as a tool to enhance teaching and learning. Inertia aside, the alternative will centre on infrastructural issues associated with ePortfolios, which in reality, are viewed as an inconvenience in the busy life of a school.

### 4.2 A policy for schools

In tandem with the identification of a common understanding discussed above ePortfolio policy exemplars should be developed for schools that are consistent with and embedded in other school policies (such as a school's Assessment and ICT AUP). Along these lines, P4 argued that ‘schools must have a policy before implementing something new’.

### 4.3 Personally owned ePortfolios

The move towards personally owned devices and ePortfolios seems to be regarded favourably for a variety of reasons. These include a reduction in the costs incurred in maintenance and the constant availability of ePortfolios to be used for varying purposes (EUfolio, 2015c). However, it is also recommended that specifications are provided by the DES and other support services on the functional requirements of an ePortfolio system (e.g. showcase, workspace, transferability). These policy specifications and standards would then allow for the decentralisation of ePortfolios to schools, while at the same time allowing for consistency of Portfolio standards within the education system.

### 4.4 Continuous professional development (CPD) for teachers and students

If ePortfolios are to become a common feature in schools, teachers will naturally require training in this area. In the case of Ireland, while education support services provide extensive ePortfolio support through online learning, seminars, school visits and the provision of ePortfolio templates it is questionable whether such support can be adequate when other external demands such as School Self Evaluation (SSE) will always be high on the agenda. Indeed, although the emerging Irish framework for mandatory professional learning referred to as COSÁN (Teaching Council, 2016) continues to be purposefully inchoate owing to contentious issues surrounding mandatory CPD it is suggested that areas such as ePortfolios play a more prominent role in future iterations of COSÁN.

Finally given other factors outside of the school, such as equity of access to broadband and the disconnect between ePortfolio functions and the Irish Curriculum, the admirably intentioned *Digital Strategy* which aims to promote the use of student-owned ePortfolios within 5 years is perhaps a little unrealistic. A more conservative forecast for the full integration of ePortfolios into teaching and learning would reflect Fullan's (2007) estimation of complex change in a system, that is, anything between 5 and 10 years.

## Declarations

### Author contribution statement

Paige Poole, Martin Brown, Gerry McNamara, Joe O'Hara, Shivaun O'Brien, Denise Burns: Conceived and designed the experiments; Performed the experiments; Analyzed and interpreted the data; Contributed reagents, materials, analysis tools or data; Wrote the paper.

### Funding statement

This research did not receive any specific grant from funding agencies in the public, commercial, or not-for-profit sectors.

### Competing interest statement

The authors declare no conflict of interest.

### Additional information

No additional information is available for this paper.

## References

[bib88] Akçıl U., Arap I. (2009). The opinions of education faculty students on learning process involving e-portfolios. Procedia Soc. Behav. Sc..

[bib93] Amarach Research (2016). Connected Futures.

[bib1] Austin R., Hunter B. (2013). ICT policy and implementation in education: cases in Canada, Northern Ireland, and Ireland. Eur. J. Educ..

[bib2] Barrett H.C. (2010). Balancing the two faces of ePortfolios. Educ. Formação Tecnol..

[bib3] Batson T. (2011). Situated learning: a theoretical frame to guide transformational change using electronic portfolio technology. Int. J. ePortfolio.

[bib4] Baylor A.L., Ritchie D. (2002). What Factors Facilitate Teacher Skill, Teacher Morale, and Perceived Student Learning in Technology-using Classrooms [Online]. http://www.motivationalexperience.com/PDF/factor.pdf.

[bib6] Black P., Wiliam D. (2010). Inside the black box. Kappan Classic.

[bib81] Borko H., Michalec P., Timmons M., Siddle J. (1997). Student teaching portfolios: a tool for promoting reflective practice. J. Teach. Educ..

[bib77] Butler D., Leahy M., Shiel G., Cosgrove J. (2013). Building Towards a Learning Society: A National Digital Strategy for Schools.

[bib8] Buzzetto-More N.A. (2007). Advanced Principles of Effective e-Learning.

[bib10] Chang C.C., Liang C., Tseng K.H., Tseng J.S. (2014). Using e-portfolios to elevate knowledge amassment among university students. Comput. Educ..

[bib90] Coe R. (2009). School improvement: reality and illusion. Br. J. Educ. Stud..

[bib14] Consortium for School Networking (2013). Rethinking Acceptable Use Policies to Enable Digital Learning: a Guide for School Districts [Online]. http://www.cosn.org/sites/default/files/pdf/Revised%20AUP%20March%202013_final.pdf.

[bib15] Cramer S.P. (1993). Navigating the assessment maze with portfolios. Clear. House.

[bib91] Creswell J.W. (2008). Research design: qualitative, quantitative, and mixed methods approaches (third ed.). [Kindle Edition 1.10.1].

[bib19] Cyprus Pedagogical Institute (2015). EUfolio: EU Classroom EPortfolios Training Booklet [Online]. http://eufolio.eu/docs/EUfolio_Training_Booklet.pdf.

[bib20] Department of Communications, Energy and Natural Resources (2015). Schools 100Mbps Project [Online]. http://www.dcenr.gov.ie/communications/en-ie/Broadband/Pages/Schools-100Mbps-Project.aspx#.

[bib21] Department of Education and Skills (1997). Schools it 2000, a Policy Framework for the New Millennium.

[bib22] Department of Education and Skills (2008). ICT in Schools: Inspectorate Evaluation Studies [Online]. https://www.education.ie/en/Publications/Inspection-Reports-Publications/Evaluation-Reports-Guidelines/ICT-in-Schools-Inspectorate-Evaluation-Studies.pdf.

[bib23] Department of Education and Skills (2015). Digital Strategy for Schools: Enhancing Teaching, Learning, and Assessment.

[bib24] Department of Interactive Media and Educational Technologies (2015). EUfolio Process Specification [Online]. https://eufolioresources.files.wordpress.com/2015/03/del10_eufolioprocessspecification_extsum_en.pdf.

[bib25] Downes R. (2012). Bring Your Own Device (BYOD) [Online]. http://www.cpaireland.ie/docs/default-source/media-and-publications/accountancy-plus/it/bring-your-own-device-(byod).pdf?sfvrsn=2.

[bib26] ElfEL (2016). ePortfolio for All [Online]. http://www.eife-l.org/activities/campaigns.

[bib27] EUfolio (2013). EUfolio Process Specification [Online]. http://mahara.eufolio.eu/artefact/file/download.php?file=39151&view=9951.

[bib28] EUfolio (2015). ePortfolio Implementation Guide for Policymakers and Practitioners [Online]. http://eufolio.eu/docs/ePortfolio_Implementation_Guide.pdf.

[bib29] EUfolio (2015). EU Classroom EPortfolios: Pilot Evaluation Results [Online]. http://eufolio.eu/docs/PilotEvaluationResults.pdf.

[bib30] EUfolio (2015). Review of Existing EPortfolio Policies and Practices [Online]. http://eufolio.eu/docs/D.8%20Policy%20Practice%20Review_short_EN.pdf.

[bib31] EUfolio (2015). EU Classroom EPortfolios: Training Booklet [Online]. http://eufolio.eu/docs/EUfolio_Training_Booklet.pdf.

[bib35] Fitch D., Peet M., Glover-Reed B., Tolman R. (2008). The use of ePortfolios in evaluating the curriculum and student learning. J. Soc. Work. Educ..

[bib37] Fullan M. (2007). The New Meaning of Educational Change.

[bib38] Gibson D., Jafari A. (2006). ePortfolio decisions and dilemmas. Handbook of Research on EPortfolios.

[bib39] Hallam G., Harper W., McCowan C., Hauville K., McAllister L., Creagh T. (2008). Australian EPortfolio Project: EPortfolio Use by university Students in Australia: Informing *Excellence in Policy and Practice*.

[bib40] Hattie J., Timperley H. (2007). The power of feedback. Rev. Educ. Res..

[bib41] Hattie J. (2008). Visible Learning: a Synthesis of over 800 Meta-analyses Relating to Achievement.

[bib43] Hislop H. (2015). Advancing Learning, Assessment and Evaluation in Irish Second Level Schools. Address to Association of Community and Comprehensive Schools Annual Convention 2015, 19 March 2015. Galway Bay Hotel, Galway [Online]. https://www.education.ie/en/Press-Events/Speeches/2015-Speeches/SP15-03-19.pdf.

[bib46] Johnston K. (2014). The Development and Implementation of ICT Policy for Schools in the Irish Post-Primary Context: a Critical Analysis.

[bib75] Klenowski V. (2000). Portfolios: promoting teaching. Assess. Educ..

[bib48] Kok I., Blignaut S. (2009). Introducing Developing Teacher-students in a Developing Context to E-portfolios [Online]. http://www.puk.ac.za/opencms/export/PUK/html/as/oo/OO_Docs_2009/e-Portfolios_NWU_Kok_Blignaut_ILearn_09_02-01-09.pdf.

[bib50] Lorenzo G., Ittelson J. (2005). An Overview of E-portfolios [Online]. https://net.educause.edu/ir/library/pdf/ELI3001.pdf.

[bib87] Love T., Cooper T. (2004). Designing online information systems for portfolio-based assessment: design criteria and heuristics. J. Inf. Technol. Educ..

[bib52] Mann K., Gordon J., MacLeod A. (2009). Reflection and reflective practice in health professions education: a systematic review. Adv. Health Sci. Educ..

[bib79] Marshal K., MacNair V. (2005). e-portfolios: Reflection, Collaboration and Sustainability in Early Teacher Education [Online]. http://bit.do/mmc2005.

[bib54] McCloud R. (2004). Using EPortfolios for Engaged Learning [Online]. https://www.ctdlc.org/Evaluation/FIPSEdocs/EngagedLearning2.pdf.

[bib55] McGhee R., Kozma R. (2001). New teacher and student roles in the technology-supported classroom. Annual Meeting of the American Educational Research Association.

[bib56] McLoughlin C., Lee J.W. (2010). Personalised and self-regulated learning in the Web 2.0 era: International exemplars of innovative pedagogy using social software. Australas. J. Educ. Technol..

[bib80] Meyer K.D., Tusin F.L. (1999). Preservice Teachers' perceptions of portfolios: process versus product. J. Teach. Educ..

[bib92] Miles M., Huberman A. (1994). Qualitative data analysis: An expanded source book.

[bib59] Miller A., O'Neill O. (2014). Supporting Successful Learning Pathways Using EPortfolio and Mobile Devices [Online]. http://dev.rtosoftware.com.au/wp-content/uploads/2011/07/2011-06-16-Australian-Flexible-Learning-Framework-supporting-Successful-learning-pathways.pdf.

[bib78] Mulkeen A. (2003). What can policy makers do to encourage integration of information and communications technology? Evidence from the Irish school system. Technol. Pedagog. Educ..

[bib60] National Council for Curriculum and Assessment (2004). Information and Communications Technology (ICT) in the Primary School Curriculum: Guidelines for Teachers [Online]. http://www.ncca.ie/en/Publications/Syllabuses_and_Guidelines/Information_and_Communications_Technology_ICT_in_the_Primary_School_Curriculum_Guidelines_for_Teachers.pdf.

[bib61] National Council for Curriculum and Assessment (2010). Towards Learning: an Overview of Senior Cycle Education [Online]. http://ncca.ie/en/Senior_Cycle/Towards_Learning_an_overview_/Towards_Learning_an_overview.pdf.

[bib62] National Council for Curriculum and Assessment (2012). Key Skills of Junior Cycle.

[bib64] Ochola J.E., Achrazoglou J., Anthony R. (2015). ePortfolio using the power of non-linear space to create and interlink a repertoire of skills essential for teaching. Electron. J. Incl. Educ..

[bib65] Patton M.Q. (2014). Qualitative Research and Evaluation Methods: Integrating Theory and Practice.

[bib66] Paulson F.L., Paulson P.R., Meyer C.A. (1991). What Makes a Portfolio a Portfolio? [Online]. http://web.stanford.edu/dept/SUSE/projects/ireport/articles/e-portfolio/what%20makes%20a%20portfolio%20a%20portfolio.pdf.

[bib83] Pelgrum W.J. (2001). Obstacles to the integration of ICT in education: results from a worldwide educational assessment. Comput. Educ..

[bib69] Professional Development Service for Teachers (2015). PDST Literacy Conference 2015 - Unlocking the Door to Learning [Online]. http://www.pdst.ie/node/5836.

[bib70] Rate N. (2008). Assessment for Learning and EPortfolios: what Are the Formative Benefits of EPortfolios? EFellow.

[bib72] Regis University (2003). Regis University Electronic Portfolio Project [Online]. http://academic.regis.edu/laap/eportfolio/basics_types.htm.

[bib73] Roberts G., Aalderink W., Cook J., Feijen M., Harvey J., Lee S., Wade V.P. (2005). Reflective learning, future thinking: digital repositories, e-portfolios, informal learning and ubiquitous computing. Research Seminar at ALT Spring Conference [Online]. http://www.immagic.com/eLibrary/ARCHIVES/GENERAL/ALT_UK/A051118R.pdf.

[bib76] Stefani L., Mason R., Pegler C. (2007). The Educational Potential of E-Portfolios: Supporting Personal Development and Reflective Learning.

[bib85] Strudler N., Wetzel K. (2005). The diffusion of electronic portfolios in Teacher education: issues of initiation and implementation. J. Res. Technol. Educ..

[bib94] Teaching Council (2016). Code of Professional Conduct for Teachers.

[bib82] Tellefsen B., Tellefesen B. (1995). Constituent orientation: theory, measurements and empirical evidence. Market Orientation.

[bib84] Theodosiadou D., Konstantinidis A. (2015). Introducing e-portfolio use to primary school pupils: response, benefits, and challenges. J. Inf. Technol. Educ.: Innovat. Pract..

[bib89] Yastibas A.E., Cepik S. (2015). Teachers' attitudes toward the use of e-portfolios in speaking classes in English language teaching and learning. Procedia Soc. Behav. Sci..

[bib86] Young D., Lipczynski K. (2007). Transferability of ePortfolios in Education: Phase One Report.

